# Temporal association patterns and dynamics of amyloid-β and tau in Alzheimer’s disease

**DOI:** 10.1007/s10654-017-0326-z

**Published:** 2017-10-25

**Authors:** Alison K. Ower, Christoforos Hadjichrysanthou, Luuk Gras, Jaap Goudsmit, Roy M. Anderson, Frank de Wolf, for the Alzheimer’s Disease Neuroimaging Initiative

**Affiliations:** 1 Department of Infectious Disease Epidemiology, School of Public Health, Imperial College, St Mary’s Campus, Norfolk Place, London, W2 1PG UK; 2 Janssen Prevention Center, Leiden, The Netherlands; 3 Amsterdam Neuroscience, Academic Medical Center, University of Amsterdam, Amsterdam, The Netherlands; 4 Department of Epidemiology, Harvard T.H. Chan School of Public Health, Boston, MA USA

**Keywords:** Biomarkers, Alzheimer’s disease, Incubation period

## Abstract

The elusive relationship between underlying pathology and clinical disease hampers diagnosis of Alzheimer’s disease (AD) and preventative intervention development. We seek to understand the relationship between two classical AD biomarkers, amyloid-β_1−42_ (Aβ_1−42_) and total-tau (t-tau), and define their trajectories across disease development, as defined by disease onset at diagnosis of mild cognitive impairment (MCI). Using longitudinal data from the Alzheimer’s Disease Neuroimaging Initiative (ADNI), we performed a correlation analysis of biomarkers CSF Aβ_1−42_ and t-tau, and longitudinal quantile analysis. Using a mixed effects model, with MCI onset as an anchor, we develop linear trajectories to describe the rate of change across disease development. These trajectories were extended through the incorporation of data from cognitively normal, healthy adults (aged 20–62 years) from the literature, to fit sigmoid curves by means of non-linear least squares estimators, to create curves encompassing the 50 years prior to MCI onset. A strong right-angled relationship between the biomarkers Aβ_1-42_ and t-tau is detected, implying a highly non-linear relationship. The rate of change of Aβ_1-42_ is correlated with the baseline concentration per quantile, reflecting a reduction in the rate of loss across disease within subjects. Regression models reveal significant amyloid loss relative to MCI onset (− 2.35 pg/mL/year), compared to minimal loss relative to AD onset (− 0.97 pg/mL/year). Tau accumulates consistently relative to MCI and AD onset, (2.05 pg/mL/year) and (2.46 pg/mL/year), respectively. The fitted amyloid curve shows peak loss of amyloid 8.06 years prior to MCI diagnosis, while t-tau exhibits peak accumulation 14.17 years following MCI diagnosis, with the upper limit not yet reached 30 years post diagnosis. Biomarker trajectories aid unbiased, objective assessment of disease progression. Quantitative trajectories are likely to be of use in clinical trial design, as they allow for a more detailed insight into the effectiveness of treatments designed to delay development of biological disease.

**Electronic supplementary material:**

The online version of this article (doi:10.1007/s10654-017-0326-z) contains supplementary material, which is available to authorized users.

## Introduction

In 2015, the World Alzheimer Report estimated there to be 46.8 million people globally living with dementia, with projections suggesting that this figure will increase to 131.5 million by 2050 [1]. Alzheimer’s disease (AD) is the most common cause of dementia, accounting for roughly two-thirds of all dementia cases [2–4]. At present, a definitive diagnosis of AD can only be made post-mortem, through a combination of clinical and histopathological evidence [5]. Prior to death, a diagnosis of probable or possible AD can be made. The former meets the criteria for dementia which specifies a worsening in cognition (amnestic and/or non-amnestic), in combination with onset over a time period of months to years [6]. The latter may present with an atypical course concomitant with disease presentation [6].

A review of clinical trials performed for potential treatments between 2002 and 2012 recorded 413 trials conducted, including both symptomatic and disease altering agents, with a 99.6% failure rate [7]. There are a number of possible explanations as to why clinical trials are failing so frequently in this disease therapy area. Late onset Alzheimer’s disease (LOAD) at present constitutes a heterogeneous condition where individuals often present with so called ‘mixed’ dementias [8]. To complicate this further, in a review of the National Alzheimer’s Coordinating Centre (NACC) database, researchers found that roughly 15% of subjects diagnosed with probable AD did not actually have AD-typical neuropathology [9], highlighting the need to specify between a biological or clinical endpoint. Further, it is likely that interventions at this late, and potentially irreversible, stage of the disease have a low probability of success if these potential drugs delay but do not reverse the development of AD pathology, as it is believed that biochemical and neuropathological changes occur in the 20 years prior to the onset of cognitive decline [7, 10–12].

The careful choice of biomarkers of disease progression can (1) facilitate understanding of the biochemical processes triggering or controlling disease development, (2) generate insight into whether or not treatments are able to modify the underlying disease pathology [13], and (3) improve the design of preventative treatment trials and in particular, the choice of end points, such that there is a larger portion of individuals enrolled present with preclinical AD-typical markers. Classical AD-biomarkers such as CSF Aβ_1−42_, total-tau (t-tau), and phosphorylated-tau (p-tau) are well-documented within the literature as being able to distinguish between AD and cognitively normal (CN) individuals [12]. Bateman et al., using the Dominantly Inherited Alzheimer’s Network (DIAN) data, suggested a temporal ordering pattern of biomarker, neurophysiological, and cognitive changes in the two and a half decades prior to expected disease onset [14]. Presently, there exists little data from which to build a data-based biomarker trajectory model, due to the long incubation period of disease, which would require an extremely large, diverse, cohort study spanning decades of sample collection. Jack et al. plotted dynamic trajectories in patients over time, of not only Aβ_1-42_ and tau, but also other neurophysiological and cognitive markers in the AD pathophysiological model [10, 15]. Shaw et al. describe the utility of threshold values for classification of individuals, with sensitivity of CSF Aβ_1-42_, t-tau, and p-tau found to be 96.4, 69.6, and 67.9%, respectively, and specificity 76.9, 92.3, and 73.1%, respectively [12]. The work presented here utilises these thresholds to describe four distinct biological phenotypes of disease, and builds upon the unique correlation of CSF Aβ_1−42_ and CSF t-tau observed by Shaw et al. to assess the continuity of the correlation longitudinally, in relation to disease progression.

We analyse the change in levels of CSF Aβ_1−42_ and t-tau across disease states, and over time, and applicability of biological profiles to diagnosis using the ADNI dataset. Utilising longitudinal biomarker trajectories anchored to MCI diagnosis, we develop two sets of novel, quantitative biological disease trajectories.

## Methods

### Dataset used

For our analysis we used the Alzheimer’s Disease Neuroimaging Initiative (ADNI) database (adni.loni.usc.edu). The ADNI was launched in 2003 as a public–private partnership, led by Principal Investigator Michael W. Weiner, MD. The primary goal of ADNI has been to test whether serial MRI, PET, other biological markers, and clinical and neuropsychological assessment can be combined to measure the progression of mild cognitive impairment (MCI) and early Alzheimer’s disease (AD). For up-to-date information, see www.adni-info.org. The dataset used was downloaded on October 31st, 2016.

Our baseline analysis is based on measurements from 1118 individuals for whom both markers, CSF Aβ_1−42_ and t-tau, were available at the baseline visit and did not have a baseline clinical diagnosis of ‘subjective memory concern’ (SMC), constituting a study population of 273 cognitively normal (CN), 272 early mild cognitive impairment (EMCI), 347 late mild cognitive impairment (LMCI), and 226 AD individuals, Online Resource 1, Table A.1. Precisely 95 individuals with a SMC diagnosis at baseline were excluded from the analyses. Within our analyses, CN and MCI (both EMCI and LMCI) individuals are divided into two classes: those with ‘progressive’ diagnostics (i.e. CN individuals who progress to MCI during follow-up, CN-P, and MCI individuals who progress to AD, MCI-P), and those who remain within the same clinical class across follow-up as at baseline. For simplicity, these individuals will be referred to as ‘non-progressors’ (NP) from this point onwards, and denoted by CN-NP and MCI-NP for CN and MCI individuals, respectively. During the longitudinal study, 431 individuals moved either from CN to MCI, or from MCI to AD, or progressed from CN to AD through MCI, while a date of MCI/AD diagnosis is known for a further 420 subjects who entered ADNI under a(n) MCI/AD diagnosis.

All analyses involving CSF data was performed using the UPENN CSF data and the adjusted concentration values in the database. Baseline CSF data for individuals originally enrolled under ADNI 1 was obtained from the UPENNBIOMK dataset, whereas the first observation in dataset UPENNBIOMK 5–8 was used for those whose original protocol was ADNI-GO or ADNI-2.

Longitudinal analyses are based on CSF samples from 185 individuals with two or more CSF samples, of both Aβ_1−42_ and t-tau. To minimise bias through batch effects and allow good longitudinal comparability of CSF concentrations, data from the UPENNBIOMK 6 dataset was used in combination with UPENNBIOMK 4 when data for that subject and/or visit was not present, as specified in ‘An Overview of the first 8 ADNI CSF Batch Analyses’ [16].

### Baseline correlation between CSF Aβ_1−42_ and t-tau: the biological phenotype classification

To assign a biological phenotype to each individual at baseline, previously defined thresholds were applied; greater than 192 pg/mL for CSF Aβ_1−42_ reflects typically CN individuals and greater than 93 pg/mL for CSF total-tau reflects concentrations of typically AD subjects [12]. Threshold values were described by Shaw et al. (2009), through evaluating the concentration of each biomarker in CSF in the period preceding death, and confirming AD pathology post-mortem [12].

### Statistical considerations for longitudinal analyses

All statistical analyses were performed in R (version 3.3.2). For the longitudinal quantile analysis, the thresholds for each of the ten quantiles were defined using the baseline visit data in isolation, before allocating all samples for an individual to the appropriate quantile group. Individual linear mixed effects models were fitted to each quantile group, without adjusting for either clinical diagnosis at the baseline visit or knowledge of progression across follow-up. Longitudinal trends of CSF Aβ_1−42_ and t-tau anchored to diagnosis of MCI or AD are quantified through mixed effect models. While we hypothesize that the trajectory of the above CSF markers from healthy adulthood across through disease development follows a sigmoid shaped curve [10, 14, 15], over the shorter time interval of the ADNI data, and in the context of high variance in measures, a linear model is determined to be a good description of the changes in these biomarkers over time. To ensure that the importance of biological phenotype on progression to MCI and AD is not overestimated due to censored follow-up, we performed two cox-proportional hazards analyses. In each model, we include the factor of biological phenotype (1—high Aβ, low t-tau; 2—low Aβ, low t-tau; 3—low Aβ, high t-tau; 4—high Aβ, high t-tau), age, and gender, with an extra variable included in the model for AD diagnosis, diagnosis at baseline (i.e. CN or MCI).

### Extrapolation of the sigmoid curve: dynamics of Aβ_1-42_ and t-tau

There are 235 individuals with Aβ_1−42_ measurements and 230 with t-tau measurements in addition to a known diagnosis date. In total, there are 157 CSF Aβ_1−42_ measurements and 157 CSF t-tau measurements at distinct time points from individuals that during the study moved to/from the MCI state. 83 CN individuals with CSF Aβ_1−42_ measurements have not moved to the MCI state during the study, and 99 individuals with CSF Aβ_1−42_ measurements have developed AD but the time since MCI is unknown. Similarly, 81 CN individuals with t-tau measurements have not moved to MCI during the study, and for 94 AD individuals with CSF t-tau measurements the time since MCI is unknown. Based on the available information in ADNI, we estimated the expected time required for a CN individual to move to the MCI state, ${\bar{\text{t}}}_{{{\text{CN}},{\text{MCI}}}}$, and the expected time required for an MCI individual to develop AD, ${\bar{\text{t}}}_{{{\text{MCI}},{\text{AD}}}}$, within the duration of the study, i.e. within 10 years. CN individuals that have not moved to the MCI state were then normally distributed before MCI (t = 0) with mean ${\bar{\text{t}}}_{{{\text{CN}},{\text{MCI}}}}$ and standard deviation ${\text{SD}}_{{{\text{CN}},{\text{MCI}}}}$. Similarly, AD individuals with the time since MCI unknown were normally distributed after t = 0 with mean ${\bar{\text{t}}}_{{{\text{MCI}},{\text{AD}}}}$ and standard deviation ${\text{SD}}_{{{\text{MCI}},{\text{AD}}}}$. Due to the short duration of the ADNI study and the old age of the participants, as well as the nature of the study, it is difficult to predict the dynamic behaviour of CSF Aβ_1-42_ and CSF t-tau during the whole course of the disease based only on this dataset. In order to shed light into the dynamics of these biomarkers across disease development, we also used available CSF Aβ_1−42_ and CSF t-tau data from Sjogren et al., a detailed cross-sectional study of healthy adults between the ages of 21 and 93 years of age [17]. The concentrations presented by Sjogren et al. were transformed from ELISA quantification values to MIA quantification values, using the general relationship presented by Toledo et al. (2015), see Supplemental Figure 1 [18]. We defined healthy individuals to be those that are at least 10 years before MCI as it is defined in ADNI. From the existing information in ADNI, we calculated the average age at the point of transition to the MCI state (t = 0) to be 72 years old. Hence, all the individuals in Sjogren et al. (2001) data that were younger than 62 years have been used in this analysis. We assumed that both CSF Aβ_1−42_ and CSF t-tau have a sigmoidal behaviour across the course of the disease. For this reason, we fitted to data a sigmoid function of the form ${\text{B}}\left( {\text{t}} \right) = {{{\text{a}}_{\text{B}} + \left( {{\text{b}}_{\text{B}} - {\text{a}}_{\text{B}} } \right)} \mathord{\left/ {\vphantom {{{\text{a}}_{\text{B}} + \left( {{\text{b}}_{\text{B}} - {\text{a}}_{\text{B}} } \right)} {\left( {1 + \exp \left( {{\text{c}}_{\text{B}} \left( {{\text{t}} - {\text{d}}_{\text{B}} } \right)} \right)} \right)}}} \right. \kern-0pt} {\left( {1 + \exp \left( {{\text{c}}_{\text{B}} \left( {{\text{t}} - {\text{d}}_{\text{B}} } \right)} \right)} \right)}}$, where ${\text{B}} \in \{ {\text{A}}\upbeta_{1 - 42} , {\text{t - tau}}\}$ and t is the time from MCI state. ${\text{a}}_{\text{B}}$ is the minimum value of B, ${\text{b}}_{\text{B}}$ is the maximum value, ${\text{c}}_{\text{B}}$ is the growth/decay rate of the exponential function, and ${\text{d}}_{\text{B}}$ is the inflection point of the function. This function was fitted to the above data using non-linear least squares. For fitting the function to CSF t-tau data, ${\text{b}}_{\text{t - tau}}$ was fixed to 300 pg/mL, which is around the maximum value that has been observed in AD individuals in ADNI. For the estimation of the prediction bound in Fig. 4a, b we used a Combined Error Model. The prediction interval indicates that there is a 95% chance that a new observation will lie within this interval given a single predictor value. For Fig. 4d, we generated data for CSF Aβ_1−42_ and CSF t-tau using the predictions (best fits, *f*) of the models and adding a measurement error/noise level given by (*a* + *b*|*f*|)*e* (combined error model), where *e* is a standard mean-zero and unit-variance (Gaussian) normal variable and *a* and *b* are parameters that have been obtained by fitting the models to data.

## Results

### Correlation between CSF Aβ1-42 and t-tau

**Fig. 1:**
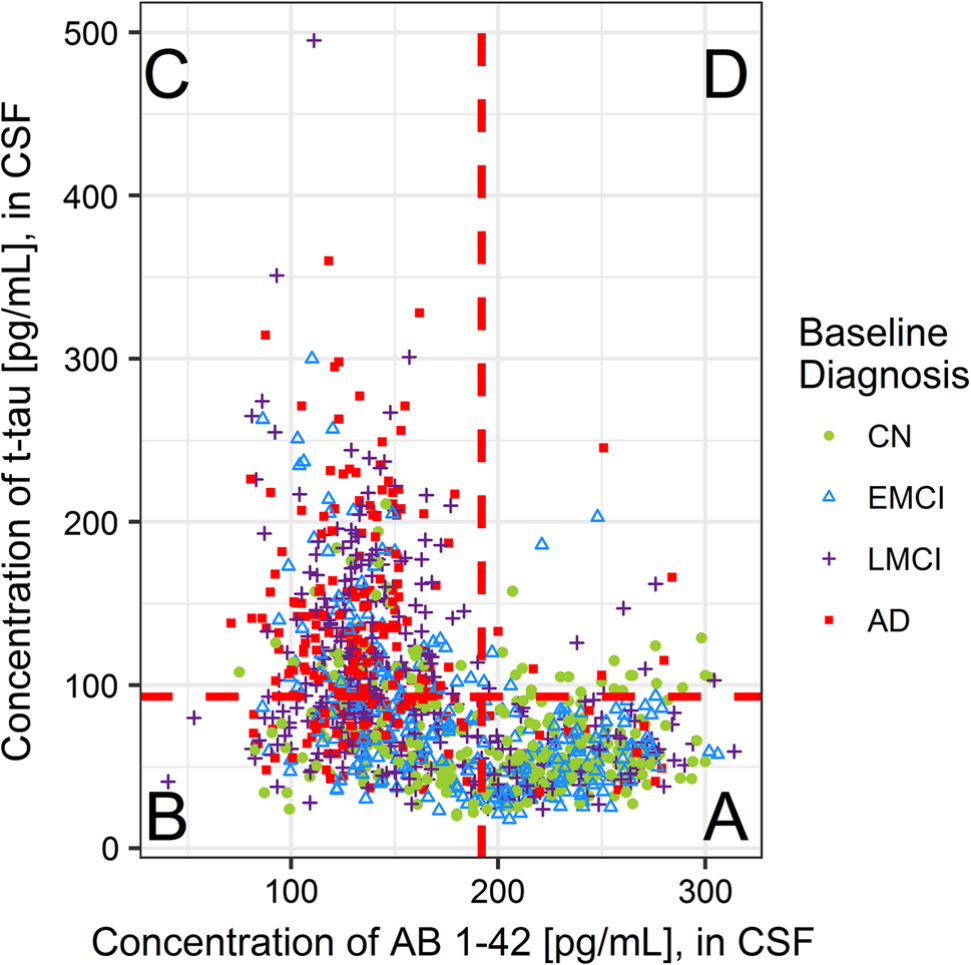
Correlation of CSF Aβ_1-42_ and CSF t-tau. Compartment A: high CSF Aβ_1−42_, low CSF t-tau (CN typical); Compartment B: low CSF Aβ_1−42_, low CSF t-tau (intermediate stage); Compartment C: low CSF Aβ_1−42_, high CSF t-tau (AD typical); Compartment D: high CSF Aβ_1−42_, high CSF t-tau (Unclassified). Colour and shape coding reflects diagnostic status at baseline (screening visit) with cognitively normal (CN) subjects in green circles, early MCI (EMCI) in blue triangles, late MCI (LMCI) in purple crosses, and Alzheimer’s disease (AD) in red squares

We observe a strong, right-angled, inverse correlation between CSF Aβ_1−42_ and CSF t-tau with individuals at the same diagnostic state clustered together, which suggests a non-linear relationship between the two variables and a mixture of sub-populations within the sample (Fig. 1). Through segmentation of CSF Aβ_1−42_ and CSF t-tau correlation plot into four compartments, utilising threshold values developed by Shaw et al. [12], four ‘stereotypical’ biological profiles of spanning AD development are constructed, enabling subjects to be described based on their clinical diagnoses as well as their biological (biomarker) profiles. Fair concordance between the clinical diagnostic group and that of the biological biomarker profile is shown in Online Resource 1, Table A.2. In particular, 50% of cognitively normal subjects present with a CN typical biological phenotype, while 64% of AD-diagnosed subjects fit the ‘AD-typical’ phenotype. The presence of CN-diagnosed individuals with an AD-typical or intermediate biological phenotype supports previous findings, which suggest that between 10 and 30% of cognitively normal elderly have evidence of amyloid deposition in their brains [19].

We hypothesize that across disease development, there is movement around the correlation. At a cross-sectional level, through a retrospective analysis, movement across disease development is inferred by the distribution of individuals in each quadrant by diagnostic progression group. The use of these five distinct groups, provides insight into the differing concordance between their biological phenotype and their diagnosed clinical class.

As expected, CN-NP are clustered in the CN-typical quadrant (53%), however, 10.8% of those who are diagnosed as clinically normal at baseline and do not progress to MCI and/or dementia, present with an AD-typical biological phenotype, as recorded in Table 1. Interestingly, the percentage of CN individuals (31.7%) who do progress during follow-up (CN-P), displaying this ‘cognitively normal’ biological phenotype at baseline, was lower compared to the 53% in those who remained cognitively normal. Unsurprisingly, the majority of subjects with progressive MCI (MCI-P) or diagnoses AD at baseline fit an AD-typical biological phenotype, 56.9 and 63.7% respectively. We observe a small percentage of individuals with this ‘unclassified’ biological phenotype—high levels of CSF Aβ_1−42_ (normal levels) with high levels of CSF t-tau (abnormal levels). 92% of clinically diagnosed AD individuals are presenting with CSF Aβ_1−42_ levels below the 192 pg/mL threshold. We hypothesize that the 8% of individuals presenting with high Aβ_1−42_ levels may be presenting with a non-AD dementia (e.g. suspected non-amyloid pathology (SNAP)), as they do not have low concentrations of amyloid. It is important to note that CN-P subjects are followed up for longer than their CN-NP counterparts, a mean 82.5 compared to 50.5 months, and median of 95.6 compared to 47.7 months. MCI-P subjects are followed up for a mean of 55.1 compared to 42.4 months. Nonetheless, biological phenotypes ‘Intermediate’ and ‘AD-typical’ are found to reflect statistically significant increased risk of progression to MCI and AD, when compared to a ‘CN typical’ phenotype, as assessed by a Cox-proportional hazards model, Online Resource 1, Table A.6 (MCI) and Table A.7 (AD).

**Table 1:** Percentage concordance between biological phenotype and diagnostic progression group, as determined by their last known diagnosis across follow-up

	N	A AD typical (%)	B Intermediate (%)	C CN typical (%)	D Unclassified (%)
CN-NP	232	10.8	30.2	53.0	6.0
CN-P	41	22.0	29.3	31.7	17.1
MCI-NP	475	26.3	30.9	40.0	2.7
MCI-P	144	56.9	33.3	9.7	0.0
AD	226	63.7	27.9	5.8	2.7

Diagnostic progression groups include: cognitively normal non-progressors (CN-NP), cognitively normal progressors (CN-P), non-progressive MCI (MCI-NP), progressive MCI (MCI-P), and Alzheimer’s disease (AD)

### ‘Dynamics’ of Aβ_1−42_ and t-tau

**Fig. 2:**
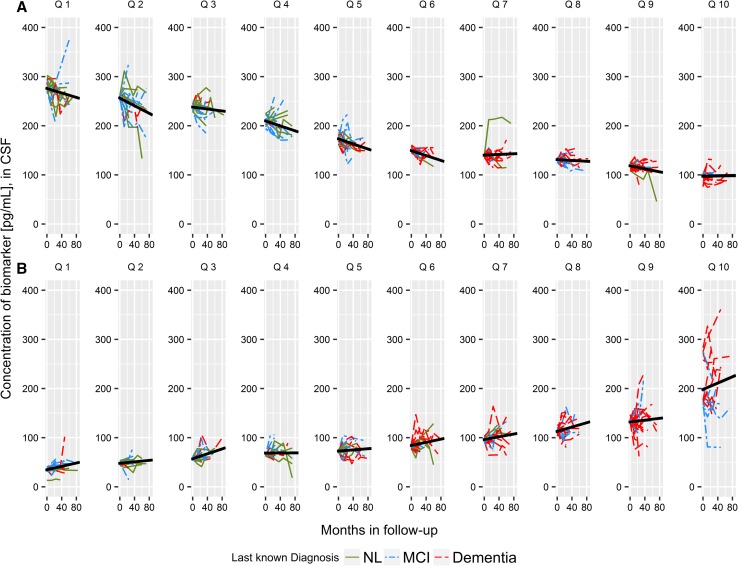
Serial measurements of **a** CSF Aβ_1−42_ and **b** CSF t-tau across follow-up, in months, stratified by quantile at baseline. Green solid line: cognitively normal diagnosis at final follow-up visit. Blue dash-dot line: MCI diagnosis at final follow-up visit. Red dashed line: an AD diagnosis at final follow-up visit. Linear mixed effect models are fit to each quantile individually, with model fits found in Online Resource 1, Table A.3 and Table A.4 for Aβ_1−42_ and t-tau respectively

In the case that biological changes reflect disease progression, regardless of cognitive status, individuals with similar biochemical marker concentrations should, theoretically, reflect similar biological disease status. From this perspective, biomarker curves can be created through a quantile analysis. When serial measurements of both Aβ_1−42_ and t-tau are visually depicted by quantile at baseline, there exists a rough, sigmoid shape of decline for Aβ_1−42_, and steady increase in t-tau as plotted in Fig. 2a, b, respectively. It is clear that AD individuals who present with characteristically low Aβ_1−42_ at baseline have reached, or reach across follow-up, a steady state of Aβ concentration. Conversely, loss of Aβ_1−42_ appears in CN and MCI individuals, who fall into the bottom 5 quantiles at baseline. Individual linear regression models reveal the greatest loss of Aβ_1−42_, unsurprisingly, within the first six quantiles, Online Resource 1, Table A.3. Whereas, in the case of t-tau, the slope across each individual linear regression model ranges from 0.004 to 0.323, without exhibiting a specific trend across the ten quantiles, Online Resource 1, Table A.4. The great variation in the concentration and trajectories of t-tau in the tenth quantile at baseline is due to the log-normal distribution of t-tau. In relation to the threshold values for AD identification, the Aβ_1−42_ threshold is crossed at quantile 5, such that all but one individual within quantile 5 has a concentration of Aβ_1−42_ less than 192 pg/mL at baseline. For t-tau, we observe this threshold being crossed at a higher quantile, quantile 7, which could be interpreted as later in the disease time-course. We observe a similar pattern when the complementary biomarker quantile grouping is used (e.g. t-tau quantile for Aβ_1−42_ concentrations), however as one would expect there is greater within quantile variation, Online Resource 1, Figures A.1 and Table A.3 (Aβ_1−42_ and t-tau by amyloid quantile), and Figure A.2 and Table A.4 (Aβ_1−42_ and t-tau by tau quantile).

### CSF trajectories in relation to MCI and AD diagnoses

**Fig. 3:**
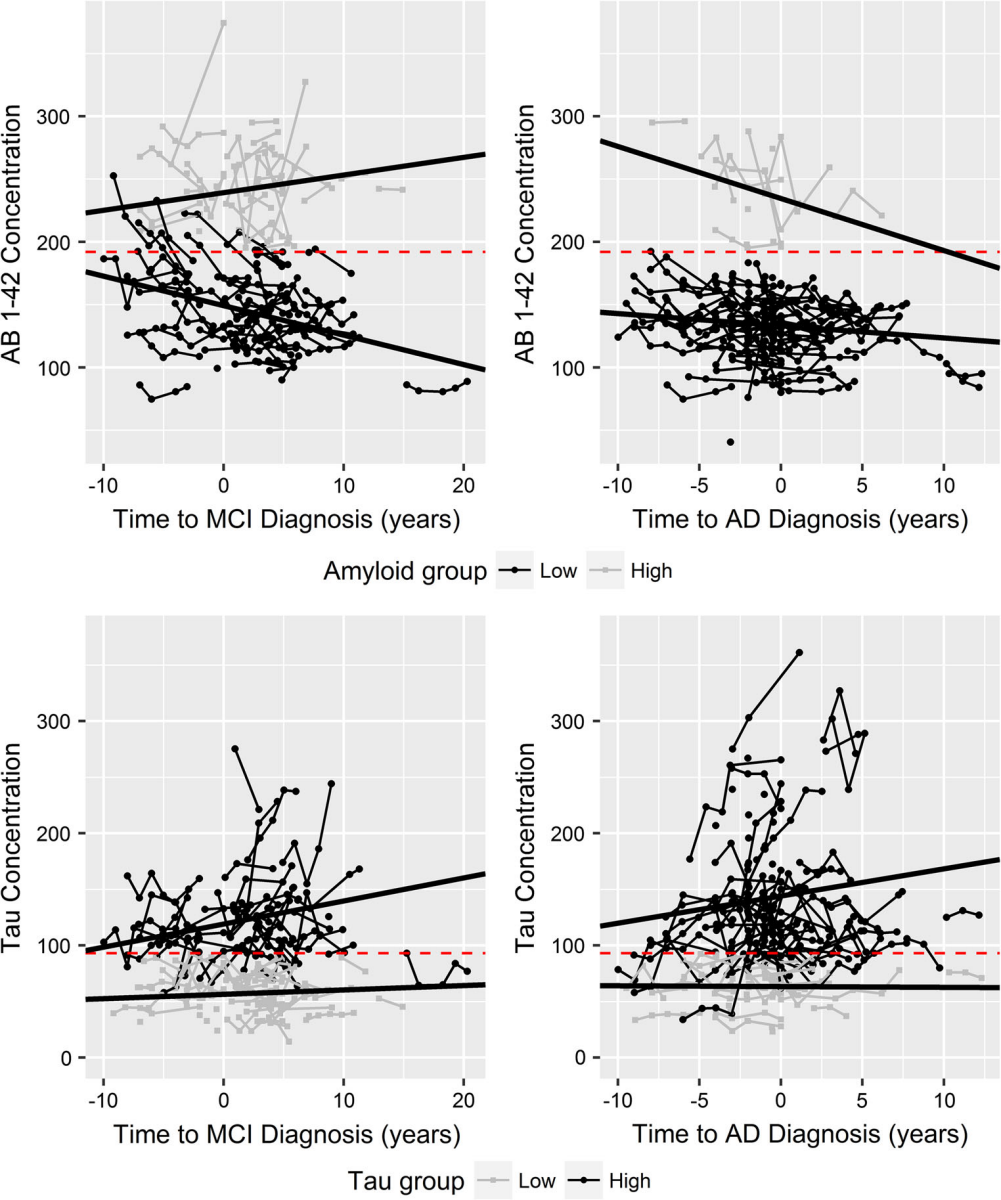
CSF longitudinal trends in relation to onset of MCI and onset of AD, in years. Subjects in black (circles) are those whose CSF concentrations reach the threshold for ‘AD typical’, as defined by Shaw et al. [12], at any point in follow-up, while those in grey (squares) remain above the threshold (Aβ_1−42_) or below the threshold (t-tau). Black lines are the fit for each group, as defined by a linear mixed effects model, with a fixed effect interaction for biomarker grouping and time, and random intercept for subject reference identification (RID). Biomarker specific threshold values are represented by red dashed lines, at 192 pg/mL and 93 pg/mL, for Aβ_1−42_ and t-tau respectively

In Fig. 3, Aβ_1−42_ and t-tau concentrations are plotted in years relative to disease onset, for both MCI diagnosis and AD diagnosis separately. Across the study population, longitudinal CSF data is available 10 years before MCI and AD diagnosis, and 10–20 years after diagnosis. For Aβ_1−42_ two distinct groups of individuals appear, those whose Aβ_1−42_ concentrations fall below the 192 pg/mL threshold at any point in follow-up, as typically observed in AD, and those for which this is never observed. The slope of the mixed effects model fit to ‘typical’ MCI individuals reflects a loss of Aβ_1−42_ (− 2.35 pg/mL/year) in the 10 years before and after MCI diagnosis, whereas when anchored to AD diagnosis, there is little loss (− 0.97 pg/mL/year) over the same period of 20 years, and relative stability across the population, Online Resource 1, Table A.5. Interestingly, the biomarker trajectory between these typical and atypical groups of individuals differs between a reference point anchored to AD diagnosis, compared to a reference point anchored to MCI diagnosis. Relative to MCI onset, those with above threshold levels of Aβ (atypical) show an increasing trajectory, whereas relative to AD diagnosis, there exists a very strong downward trend, with a much greater slope (approximately − 4.0 pg/mL/year) than what is observed for AD-typical levels across the same time-frame. For t-tau, similar groupings have been developed, however these do not reflect the strong grouping seen for Aβ_1−42_. As one would expect, in biologically typical individuals, the accumulation in CSF t-tau relative to MCI onset is less than that relative to AD onset, 2.05 pg/mL/year compared to 2.46 pg/mL/year. Individuals presenting with t-tau concentrations consistently below the 93 pg/mL threshold across follow-up, present with slopes approaching zero, 0.36 and − 0.06 pg/mL/y, for MCI-anchored and AD-anchored, respectively.

**Fig. 4:**
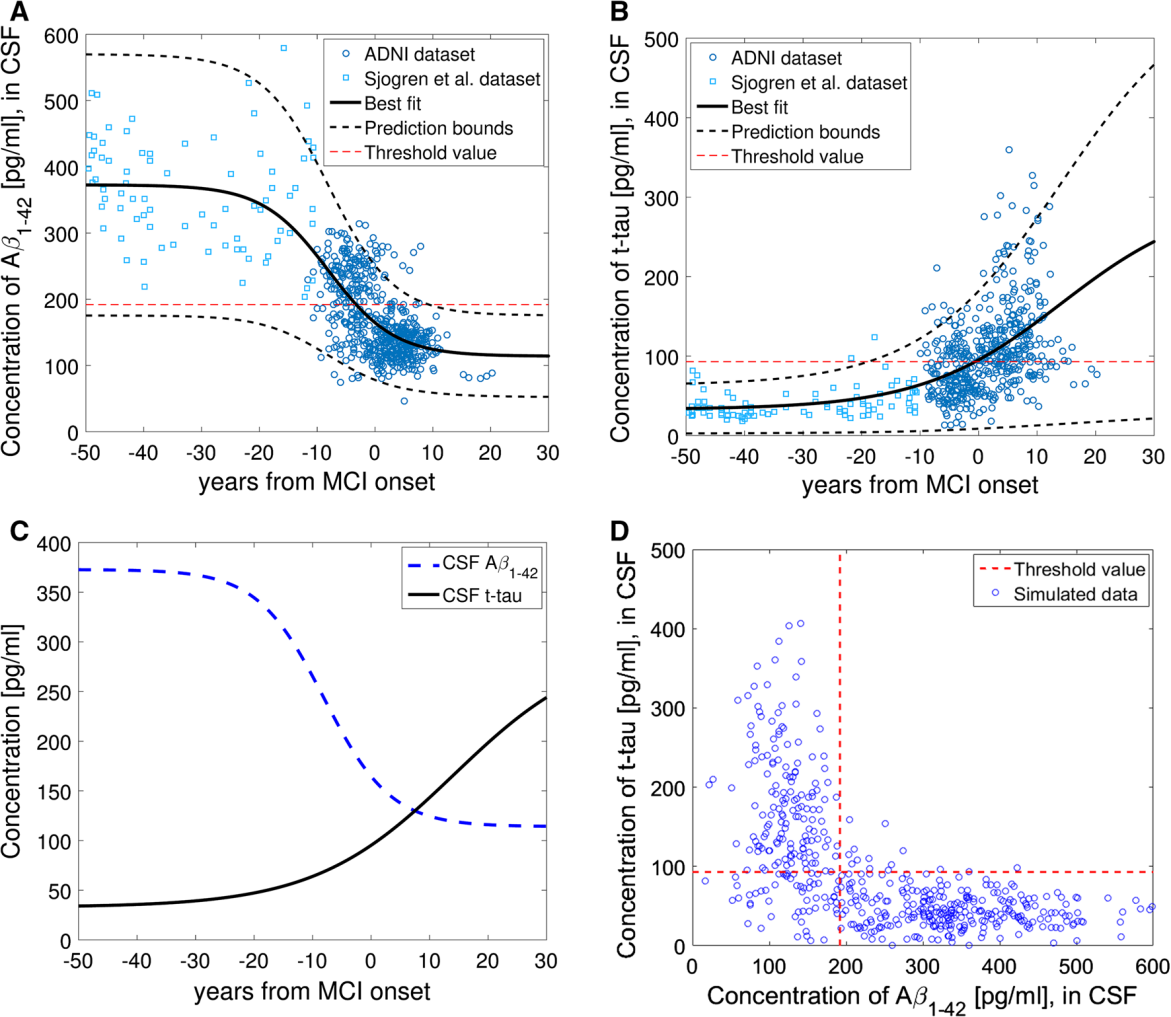
Best fit of the sigmoid function to ADNI (blue circles) and Sjogren et al. [20] data (cyan squares) of **a** CSF Aβ_1−42_ and **b** CSF t-tau, levels before and after MCI onset (t = 0) The prediction interval indicates that there is a 95% chance that a new observation will lie within this interval. **c** Best fits for CSF Aβ_1−42_ and t-tau. **d** Correlation of t-tau and Aβ_1−42_ based on data that has been simulated using the predictions in **a** and **b**. The threshold values are those defined by Shaw et al. [12]

To expand upon the linear biomarker fits, data from healthy individuals, aged 20–62, was incorporated with the ADNI data relative to MCI diagnosis, such that there is data which can be used to inform a sigmoid curve from 50 years prior to diagnosis of MCI and 20 years following diagnosis. After fitting the models to data, we observe levels of Aβ_1−42_ in young, healthy individuals of 372 pg/mL (Fig. 4a) and 32.87 pg/mL for t-tau (Fig. 4b). For CSF Aβ_1−42_, Fig. 4a, we obtained the following parameter estimates for the sigmoid function: ${\text{a}}_{{{\text{A}\upbeta}_{1 - 42} }} = 114.07$, with 95% confidence interval ${\text{CI}} = \left( {98.92, 129.22} \right)$, ${\text{b}}_{{{\text{A}\upbeta}_{1 - 42} }} = 372.79$ with ${\text{CI}} = \left( {345.46, 400.11} \right)$, ${\text{c}}_{{{\text{A}\upbeta}_{1 - 42} }} = 0.18$ with ${\text{CI}} = \left( {0.13, 0.22} \right)$ and ${\text{d}}_{{{\text{A}\upbeta}_{1 - 42} }} = - 8.06$ with ${\text{CI}} = \left( { - 9.60, - 6.51} \right)$. For CSF t-tau, Fig. 4b, we obtained: ${\text{a}}_{{{\text{t}} - {\text{tau}}}} = 32.87$ with ${\text{CI}} = \left( {27.13, 38.61} \right)$, ${\text{c}}_{{{\text{t}} - {\text{tau}}}} = - 0.084$ with ${\text{CI}} = \left( { - 0.10, - 0.067} \right)$ and ${\text{d}}_{{{\text{t}} - {\text{tau}}}} = 14.17$ with ${\text{CI}} = \left( {11.83, 16.51} \right)$. The two fitted sigmoid curves are shown together, Fig. 4c, with the point of inflection of Aβ_1−42_ at 8.06 years prior to MCI, and that of t-tau 14.17 years after MCI diagnosis. CSF Aβ_1−42_ loss stabilises to a steady state of 114.07 pg/mL in the decade following MCI diagnosis, while the steady state of t-tau has not yet been reached 30 years following diagnosis. The estimates of the expected times have been produced using Monte Carlo simulations: ${\bar{\text{t}}}_{{{\text{CN}},{\text{MCI}}}} = 4.64$ years with standard deviation ${\text{SD}}_{{{\text{CN}},{\text{MCI}}}} = 2.87$, and ${\bar{\text{t}}}_{{{\text{MCI}},{\text{AD}}}} = 4.13$ years with ${\text{SD}}_{{{\text{MCI}},{\text{AD}}}} = 2.81$. When data is simulated, an inverse correlation between Aβ_1−42_ and t-tau is demonstrated, similar to that observed in the ADNI data at baseline (Fig. 1), see Fig. 4d. Although we observe a temporal lag between the loss of CSF Aβ_1-42_ and consequent accumulation of t-tau, in the presence of a tight correlation, it is important to note that correlation does not necessarily reflect causation, with regards to biomarker deregulation and consequent pathological impacts.

## Discussion

Using the ADNI dataset we considered correlations between the key biochemical markers CSF Aβ_1−42_ and t-tau. We observed two patterns of biomarker trajectories. The first representing Aβ_1−42_ depletion in the CSF through the onset of preclinical dementia, which occurs prior to a second trajectory reflecting an accumulation in t-tau. The shape of the inverse and highly non-linear correlation between these two biomarkers suggests that amyloid may reach a critical concentration, which triggers tau accumulation. A second determinant may be necessary in combination with the presence of amyloid plaques to trigger tau conversion, a hypothesis which is supported by the molecular work of Li et al. [20] in mice, which demonstrates that amyloid is necessary, but not sufficient, for pathological conversion of tau.

The rate of change in Aβ_1−42_ and t-tau across ADNI follow-up supports the temporal ordering of biomarker trajectory curves previously described by Jack et al. [10, 15]. The longitudinal quantile analysis suggests that AD individuals in the ADNI population have already reached their plateau in amyloid-accumulation, whereas cognitively normal individuals, particularly those for whom we do not see progression to MCI across follow-up, appear to be at an early stage on the disease progression spectrum, in terms of Aβ_1−42_ loss in CSF. However, these curves of biomarker concentrations across disease development do not encompass the entire disease time-course, nor do they use continuous time. Additionally, subjects within a disease class (e.g. MCI) are likely to present with differing MCI severities at baseline, such that one MCI subject will be more diseased than another within the same class. Previous studies in the literature have performed extensive statistical analyses using time (t) relative to study baseline (t = 0) to describe biomarker trajectories [21], or attempted to circumvent the issues listed above by developing a pseudo-time scale based on cognitive decline and disease status [22, 23]. Taking together these limitations, another, and likely more instructive method to analyse change across disease, is to anchor to time of diagnosis, be that MCI or AD, and build the curves in either direction. This approach has been used by Bateman et al. [14]. and Thordardottir et al. [24] in populations with autosomal dominant familial AD mutations. Developing trajectories based on a real timescale, relative to a cognitively based diagnosis such as MCI onset, as opposed to a relative or pseudo scale, (1) allows for greater ease of use and interpretation by clinicians who are conveying biomarkers results to the patient or family members, and (2) accounts for the common discrepancy between AD-specific pathology (low CSF Aβ and high t-tau) and impaired cognition.

Building upon the trajectories of Aβ_1−42_ and t-tau relative to MCI onset from individuals in ADNI, Figs. 3, we extrapolated back using data from the literature related to both estimated incubation times and ‘normal’ biomarkers levels from healthy adults across adulthood, Fig. 4. With regards to incubation period, previous work by Bateman et al., in the DIAN study show that trajectories for amyloid and tau in CSF begin to deviate from stable levels in the 20–25 years prior to diagnosis [14], similar to the 25 years observed in our findings. The fitted sigmoid function to both ADNI and Sjogren et al. data depicts a defined sigmoid behaviour for both Aβ_1−42_ (Fig. 4a) and t-tau (Fig. 4b), with the upper threshold of the t-tau curve not yet reached after 30 years post-MCI onset. Quantitative functions such as these, which include confidence bounds, allow for the potential to interpolate diagnosis times and make educated predictions about future progression to MCI. Similar results are presented by Maia et al. [25] in an animal model of disease progression, who observed comparable temporal ordering in alterations in biomarker concentrations in mice. As hypothesized, and previously shown in the literature, the alterations in CSF Aβ_1−42_ occur prior to that of t-tau. The increase in t-tau is thought to be due to leakage from damaged neurons into the CSF, upon neurofibrillary tangle formation and neuronal degradation [26, 27], while it is believed that the decreased concentrations of Aβ_1−42_ in CSF may be due to the accumulation of Aβ_1−42_ in amyloid plaques [28, 29]. There is also reason to believe that there is an age, as well as a disease component, to tau concentrations. An increase in CSF t-tau with age has been described in the literature [17, 30], with Sjogren et al. [17] observing the levels of t-tau increasing across age in healthy aging adults; levels of t-tau appear relatively stable up to the age of 50, before increasing in an exponential manner.

It is important to note the limitations of this approach and the data that we have used. Foremost, particularly in the case of t-tau, there are multiple non-linear functions that could be fitted to this data. We believe that, due to the body of literature encompassing sigmoid behaviour of biomarkers in AD, together with the aspect of death as an endpoint, a sigmoid function was most appropriate. The ‘right angled’ nature of the phase plot of the two variables also provides strong support for a sigmoidal non-linear relationship with a steep inflection after a period of stability. It is important to note that the functions shown in Fig. 4 encompass 50 years prior to MCI onset and 30 years following. Considering an average life expectancy of 80 years and a hypothetical age of onset of 70 years, it is unlikely that individuals reach this outer boundary. Within both the data from ADNI subjects and Sjogren et al., there exists a great degree of variation in the concentration of both Aβ_1−42_ and t-tau for any given age grouping. We must also recognise that the data comes from two different sources, in two different countries (ADNI: USA; Sjogren: Sweden) and were quantified using different methods. While parsing together data from two studies is not ideal, there is currently a lack of longitudinal studies that span the course of young adulthood into old age where the disease course can be observed within subjects. This is understandable given such studies would be extremely costly and be a great burden on subjects, in terms of their time and the invasive procedures they must undergo. To best observe and compare biological trajectories across the course of disease in cohorts worldwide, both healthy and diseased, there is a need for standardised quantification methods.

Biomarkers may provide an unbiased, objective picture of underlying disease pathology, provided more attention is given to the standardisation of measurement procedures and the precise recording of measurement errors. The development of disease-specific marker trajectories are of particular interest in terms of clinical applicability, as they have the potential shed light on the state of AD pathology in relation to expected onset of clinical disease. As the field has a particular aim at developing preventative treatments markers of disease, and the temporal relationship of these markers to disease onset, are crucial to knowing who to treat and when to treat.

While classical biomarkers such as CSF Aβ_1−42_ and t-tau are able to distinguish between cognitively normal and AD individuals with fair sensitivity, there remains significant discrepancies between clinical diagnosis and biological phenotype. We presented hypothetical trajectories of CSF Aβ_1−42_ and t-tau across disease course, anchoring to the onset of MCI, utilising longitudinal data from either diseased individuals who develop or present with MCI in ADNI, and cross-sectional data from healthy young adults. The curves developed provide the potential for estimation of MCI onset within a cognitively normal, aging population, facilitating the detection of preclinical AD, and enabling a better choice of subjects that should participate in a clinical trial, maximising the chances of success of potential treatments.

## Electronic supplementary material

Below is the link to the electronic supplementary material.

Supplementary material 1 (DOCX 115 kb)
