# Supplementary material for: Temporal association patterns and dynamics of amyloid-β and tau in Alzheimer’s disease
Source: Eur J Epidemiol. 2017 Oct 25;33(7):657–66. doi: 10.1007/s10654-017-0326-z (PMC6061138; doi:10.1007/s10654-017-0326-z)
Supplement: Supplementary file 1 — Supplementary material 1 (DOCX 115 kb) [file 10654_2017_326_MOESM1_ESM.docx]

**Supplementary Tables and Figures**

Table A.1 Baseline demographics and disease markers across the four baseline diagnosis groups: cognitively normal (CN), early MCI (EMCI), late MCI (LMCI), and Alzheimer’s disease (AD). Demographic characteristics include: gender, age, apolipoprotein E (ApoE) positivity, educational attainment, race, and ethnicity. Disease markers include: CSF Aβ_1-42_, CSF t-tau, fluorodeoxyglucose (FDG)-positron emission tomography (PET), hippocampal volume, Alzheimer’s Disease Assessment Scale- cognitive subscale (ADAScog), and the Mini Mental State Examination (MMSE) score.

|  |  | **CN** | **EMCI** | **LMCI** | **AD** |
| --- | --- | --- | --- | --- | --- |
| **Number of Subjects** |  | 273 | 272 | 347 | 226 |
| **Gender Distribution** | (%) Male | 49.82 | 56.25 | 60.81 | 58.14 |
|  | (%) Female | 50.18 | 43.75 | 39.19 | 41.59 |
| **Age** | Mean years, (SD) | 74.36 (5.97) | 71.13 (7.37) | 73.41 (7.53) | 74.81 (8.13) |
| **ApoE-4 Allele** | E4 -/- (%) | 74.36 | 57.72 | 45.24 | 32.74 |
|  | E4 +/- (%) | 22.71 | 35.29 | 42.07 | 46.46 |
|  | E4 +/+ (%) | 2.93 | 6.99 | 12.68 | 20.8 |
| **Educational Attainment** | Mean years, (SD) | 16.26 (2.67) | 15.94 (2.65) | 16.15 (2.85) | 15.44 (2.97) |
| **Race** | White (%) | 89.74 | 92.65 | 95.1 | 95.58 |
|  | Black (%) | 7.69 | 2.21 | 2.59 | 2.21 |
|  | Asian (%) | 1.47 | 1.47 | 1.73 | 1.77 |
| **Ethnicity** | Non-Hispanic (%) | 95.97 | 95.22 | 97.98 | 96.46 |
|  | Hispanic/Latino (%) | 3.66 | 4.41 | 1.73 | 3.1 |
| **Biochemical Markers** | Mean CSF AB_1-42_, pg/mL, (SD) | 199.11 (52.92) | 184.87 (51.05) | 161.45 (52.81) | 139.94 (39.42) |
|  | Mean CSF t-tau, pg/mL, (SD) | 68.88 (32.54) | 76.23 (47.46) | 102.3 (58.62) | 127.48 (61.82) |
| **Neurophysiological** | Mean FDG-PET, SUV, (SD) | 1.31 (0.11) | 1.29 (0.12) | 1.22 (0.14) | 1.07 (0.14) |
|  | Mean Normalised Hippocampal volume, mm^3^, (SD) | 7439.55 (765.95) | 7295.82 (997.04) | 6461.89 (1094.97) | 5845.64 (974.43) |
| **Cognitive Scores** | Mean ADAScog-11 score, (SD) | 6.09 (3.05) | 7.88 (3.46) | 11.65 (4.7) | 19.66 (6.78) |
|  | Mean MMSE score, (SD) | 29.06 (1.15) | 28.35 (1.59) | 27.21 (1.84) | 23.33 (1.99) |

Table A.2. Percentage concordance between biological phenotype at baseline and the four baseline diagnosis groups: cognitively normal (CN), early MCI (EMCI), late MCI (LMCI), and Alzheimer’s disease (AD). 1116 subjects with both CSF Aβ_1-42_ and t-tau concentrations at their baseline visit are included.

|  |  | Biological phenotype | | | |
| --- | --- | --- | --- | --- | --- |
| Clinical diagnosis | N | AD Typical (%) | Intermediate (%) | CN Typical (%) | Unclassified (%) |
| CN | 273 | 12.5 | 30.0 | 49.8 | 7.7 |
| EMCI | 272 | 21.3 | 31.2 | 45.2 | 2.2 |
| LMCI | 347 | 42.9 | 31.7 | 23.3 | 2.0 |
| AD | 226 | 63.7 | 27.9 | 5.8 | 2.7 |

Table A.3. Linear mixed model fits for Aβ_1-42_ in CSF.

|  | AB_Concentration ~ Month + (1\|factor(Subject_ID) | | | | | | | |  | AB_Concentration ~ Month + (1\|factor(Subject_ID) | | | | | | | |
| --- | --- | --- | --- | --- | --- | --- | --- | --- | --- | --- | --- | --- | --- | --- | --- | --- | --- |
| **Amyloid Quantile** | Intercept | SE (int) | p (int) | Slope | SE (slope) | p (slope) | R^2^ marginal | R^2^ conditional | **Tau Quantile** | Intercept | SE (int) | p (int) | Slope | SE (slope) | p (slope) | R^2^ marginal | R^2^ conditional |
| **1** | 275.85 | 4.697 | 0 | -0.226 | 0.142 | 0.119 | 0.034 | 0.163 | **1** | 216.124 | 10.114 | 0 | -0.204 | 0.12 | 0.097 | 0.01 | 0.829 |
| **2** | 256.222 | 5.578 | 0 | -0.387 | 0.128 | 0.004 | 0.094 | 0.38 | **2** | 226.394 | 10.625 | 0 | -0.268 | 0.102 | 0.012 | 0.014 | 0.906 |
| **3** | 238.043 | 3.532 | 0 | -0.099 | 0.1 | 0.331 | 0.013 | 0.356 | **3** | 225.795 | 12.847 | 0 | -0.304 | 0.105 | 0.006 | 0.011 | 0.932 |
| **4** | 209.715 | 3.85 | 0 | -0.251 | 0.097 | 0.013 | 0.079 | 0.353 | **4** | 182.34 | 13.127 | 0 | -0.279 | 0.117 | 0.021 | 0.011 | 0.902 |
| **5** | 173.148 | 3.227 | 0 | -0.256 | 0.073 | 0.001 | 0.096 | 0.496 | **5** | 206.087 | 14.268 | 0 | -0.154 | 0.11 | 0.17 | 0.003 | 0.916 |
| **6** | 149.327 | 1.798 | 0 | -0.249 | 0.062 | 0 | 0.195 | 0.329 | **6** | 157.664 | 12.466 | 0 | -0.249 | 0.106 | 0.023 | 0.009 | 0.913 |
| **7** | 140.177 | 4.023 | 0 | 0.036 | 0.076 | 0.637 | 0.001 | 0.588 | **7** | 158.777 | 14.575 | 0 | 0.001 | 0.101 | 0.99 | 0 | 0.934 |
| **8** | 131.142 | 2.009 | 0 | -0.042 | 0.044 | 0.344 | 0.008 | 0.452 | **8** | 145.719 | 11.701 | 0 | -0.168 | 0.065 | 0.013 | 0.003 | 0.973 |
| **9** | 118.425 | 2.403 | 0 | -0.152 | 0.063 | 0.018 | 0.06 | 0.332 | **9** | 134.546 | 4.593 | 0 | -0.11 | 0.067 | 0.106 | 0.009 | 0.794 |
| **10** | 97.555 | 2.518 | 0 | 0.014 | 0.068 | 0.837 | 0.001 | 0.402 | **10** | 130.437 | 4.238 | 0 | -0.059 | 0.052 | 0.269 | 0.004 | 0.852 |

Table A.4. Linear mixed model fits for total-tau (t-tau) in CSF.

|  | Tau_Concentration ~ Month + (1\|factor(Subject_ID) | | | | | | |  |  | Tau_Concentration ~ Month + (1\|factor(Subject_ID) | | | | | | | |
| --- | --- | --- | --- | --- | --- | --- | --- | --- | --- | --- | --- | --- | --- | --- | --- | --- | --- |
| **Tau Quantile** | Intercept | SE (int) | p (int) | Slope | SE (slope) | p (slope) | R^2^ marginal | R^2^ conditional | **Amyloid Quantile** | Intercept | SE (int) | p (int) | Slope | SE (slope) | p (slope) | R^2^ marginal | R^2^ conditional |
| **1** | 34.839 | 2.468 | 0 | 0.17 | 0.06 | 0.007 | 0.085 | 0.42 | **1** | 66.696 | 5.647 | 0 | 0.134 | 5.647 | 0.03 | 0.012 | 0.876 |
| **2** | 47.861 | 1.793 | 0 | 0.076 | 0.051 | 0.139 | 0.031 | 0.321 | **2** | 59.546 | 3.275 | 0 | -0.023 | 3.275 | 0.684 | 0.001 | 0.609 |
| **3** | 57.201 | 2.027 | 0 | 0.251 | 0.066 | 0 | 0.168 | 0.285 | **3** | 60.083 | 6.122 | 0 | 0.072 | 6.122 | 0.21 | 0.003 | 0.921 |
| **4** | 68.724 | 2.632 | 0 | 0.004 | 0.076 | 0.96 | 0 | 0.18 | **4** | 55.752 | 5.707 | 0 | 0.188 | 5.707 | 0.001 | 0.028 | 0.893 |
| **5** | 72.761 | 2.434 | 0 | 0.059 | 0.056 | 0.293 | 0.011 | 0.384 | **5** | 86.098 | 8.353 | 0 | 0.222 | 8.353 | 0.027 | 0.014 | 0.831 |
| **6** | 84.349 | 3.125 | 0 | 0.158 | 0.083 | 0.063 | 0.041 | 0.306 | **6** | 109.761 | 12.803 | 0 | 0.161 | 12.803 | 0.134 | 0.003 | 0.944 |
| **7** | 96.204 | 3.369 | 0 | 0.14 | 0.082 | 0.093 | 0.032 | 0.327 | **7** | 121.929 | 13.277 | 0 | 0.173 | 13.277 | 0.135 | 0.003 | 0.912 |
| **8** | 112.894 | 3.468 | 0 | 0.22 | 0.103 | 0.037 | 0.05 | 0.378 | **8** | 111.534 | 11.827 | 0 | 0.139 | 11.827 | 0.24 | 0.003 | 0.868 |
| **9** | 132.02 | 5.177 | 0 | 0.093 | 0.157 | 0.558 | 0.004 | 0.216 | **9** | 115.069 | 13.22 | 0 | 0.251 | 13.22 | 0.083 | 0.007 | 0.861 |
| **10** | 198.151 | 13.677 | 0 | 0.323 | 0.191 | 0.097 | 0.01 | 0.814 | **10** | 124.963 | 14.832 | 0 | 0.06 | 14.832 | 0.733 | 0 | 0.902 |

Figure A.1. Serial measurements of CSF Aβ_1-42_ (red) and t-tau (blue) across follow-up, in months, stratified by Aβ_1-42_ quantile at baseline.


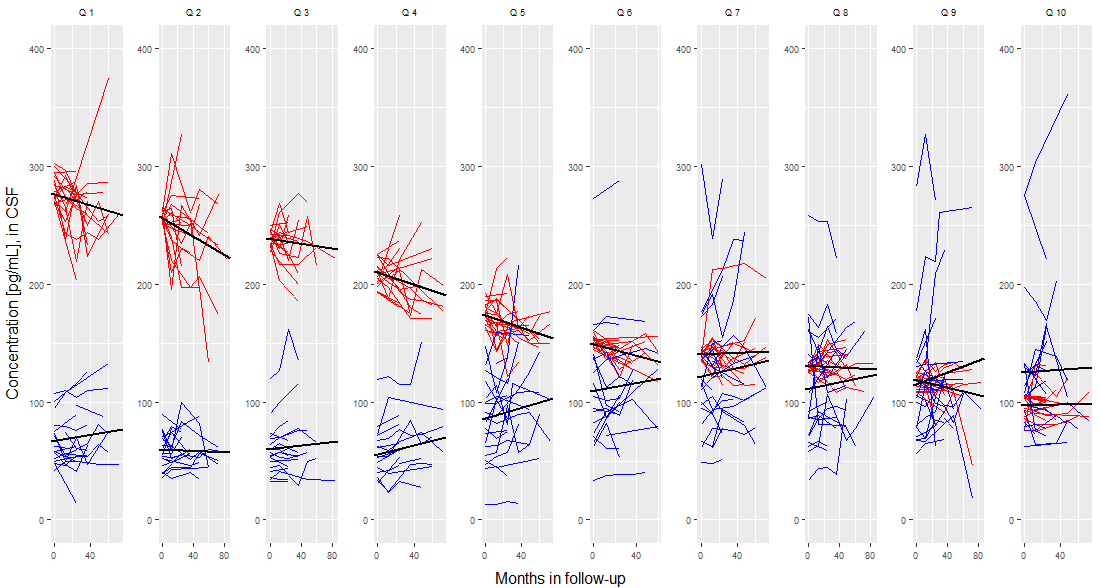


Figure A.2. Serial measurements of CSF Aβ_1-42_ (red) and t-tau (blue) across follow-up, in months, stratified by t-tau quantile at baseline.


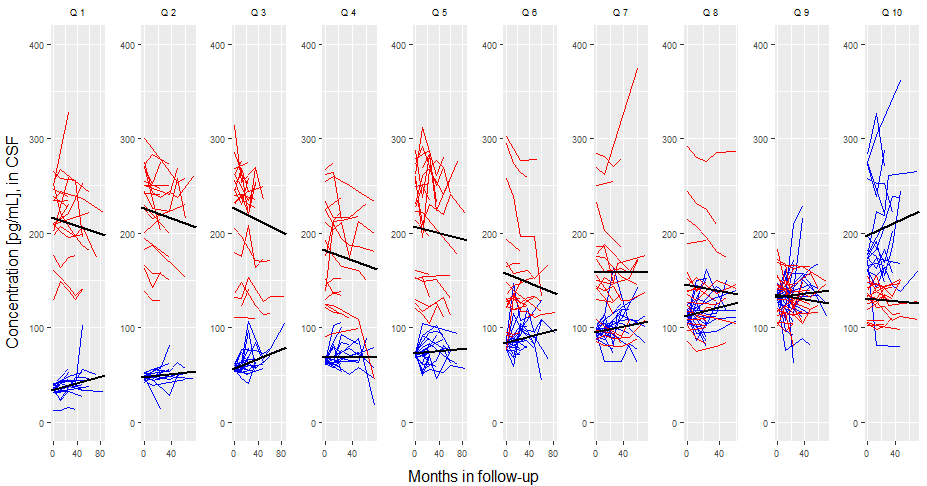


Table A.5 corresponding linear mixed model fits for Figure 3

| **AB_Concentration ~ Time_to_MCI*as.factor(High_AB) + (1\|as.factor(Subject_ID))** | | | |
| --- | --- | --- | --- |
|  | Estimate | SE | p_value |
| Intercept | 149.263 | 3.934 | 0 |
| Time_to_MCI | -2.349 | 0.461 | 0 |
| factor(High_AB) - High | 89.922 | 6.718 | 0 |
| Time_to_MCI : factorHigh_AB)High | 3.754 | 0.917 | 0 |
| R2m (Marginal): 0.662; R2c (Conditional): 0.936 |  |  |  |
| **AB_Concentration ~ Time_to_AD*as.factor(High_AB) + (1\|as.factor(Subject_ID))** | | | |
|  | Estimate | SE | p_value |
| Intercept | 133.177 | 2.114 | 0 |
| Time_to_AD : factor(High_AB)High | -0.971 | 0.336 | 0.004133 |
| factor(High_AB)High | 101.335 | 7.371 | 0 |
| Time_to_AD : factor(High_AB)High | -3.174 | 1.098 | 0.004131 |
| R2m (Marginal): 0.613; R2c (Conditional): 0.933 |  |  |  |
| **Tau_Concentration ~ Time_to_MCI*factor(Low_Tau) + (1\|as.factor(Subject_ID))** | | | |
|  | Estimate | SE | p_value |
| Intercept | 56.419 | 4.012 | 0 |
| Time_MCI | 0.39 | 0.61 | 0.523 |
| as.factor(Low_Tau)High | 62.695 | 5.894 | 0 |
| Time_MCI:as.factor(Low_Tau)High | 1.662 | 0.805 | 0.040 |
| R2m (Marginal): 0.567; R2c (Conditional): 0.898 | | | |
| **Tau_Concentration ~ Time_to_AD*factor(Low_Tau) + (1\|as.factor(Subject_ID))** | | | |
|  | Estimate | SE | p_value |
| Intercept | 63.572 | 7.203 | 3.55E-15 |
| Time_AD | -0.029 | 1.079 | 0.978 |
| as.factor(Low_Tau)High | 80.447 | 8.683 | 4.44E-16 |
| Time_AD:as.factor(Low_Tau)High | 2.489 | 1.279 | 0.053 |
| R2m (Marginal): 0.379; R2c (Conditional): 0.913 | | | |

Table A.6. Cox-proportional hazards model, progression to MCI diagnosis

| **coxph(formula = Surv(Time, Progress_to_MCI) ~ as.factor(Biological_Phenotype) + Age + Gender)**  n = 115, number of events = 34 | | | | | | | |
| --- | --- | --- | --- | --- | --- | --- | --- |
|  | Coef | Exp(coef) | Lower .95 | Upper .95 | Se (coef) | z | Pr(>\|z\|) |
| Biological Phenotype (Intermediate) | 1.08570 | 2.96150 | 1.1688 | 7.504 | 0.47438 | 2.289 | 0.02210 |
| Biological Phenotype (AD Typical) | 1.22119 | 3.39122 | 1.3713 | 8.387 | 0.46197 | 2.643 | 0.00821 |
| Biological Phenotype (Unclassified) | 0.83226 | 2.29851 | 0.6058 | 8.721 | 0.68036 | 1.223 | 0.22123 |
| Age | 0.03586 | 1.03651 | 0.9649 | 1.113 | 0.03654 | 0.981 | 0.32641 |
| Gender  (female) | -0.17214 | 0.84186 | 0.4243 | 1.671 | 0.34963 | -0.492 | 0.62248 |

Table A.7. Cox-proportional hazards model, progression to AD diagnosis

| **coxph(formula = Surv(Time, Progress_to _AD) ~ as.factor(Biological_Phenotype) + Age + Gender +   Baseline_Diagnosis)**  n = 389, number of events = 115 | | | | | | | |
| --- | --- | --- | --- | --- | --- | --- | --- |
|  | Coef | Exp(coef) | Lower .95 | Upper .95 | Se (coef) | z | Pr(>\|z\|) |
| Biological Phenotype (Intermediate) | 1.393e+00 | 4.028e+00 | 2.0287 | 7.999 | 3.500e-01 | 3.981 | 6.85e-05 |
| Biological Phenotype (AD Typical) | 2.027e+00 | 7.589e+00 | 4.1097 | 14.015 | 3.130e-01 | 6.476 | 9.42e-11 |
| Biological Phenotype (Unclassified) | -1.533e+01 | 2.208e-07 | 0.0000 | Inf | 2.446e+03 | -0.006 | 0.995 |
| Age | 2.872e-03 | 1.003e+00 | 0.9751 | 1.031 | 1.434e-02 | 0.200 | 0.841 |
| Gender  (female) | -4.494e-02 | 9.561e-01 | 0.6432 | 1.421 | 2.022e-01 | -0.222 | 0.824 |
| Baseline Diagnosis  (MCI) | .986e+00 | 7.285e+00 | 3.7077 | 14.315 | 13.446e-01 | 5.762 | 8.30e-09 |
